# Harvard

**Published:** 1896-08

**Authors:** 


### AMONG THE COLLEGES.

THE establishment of a department of comparative pathology at Harvard University, the endowment of a professorship in the work, and the selection of so well-equipped a man as Professor Theobald Smith means much to the future veterinary students of Harvard, and will afford them knowledge of a high and invaluable character.

I. D. RAWLINGS, M.S., M.D., Professor of Bacteriology at the Northwestern University Medical School, has accepted the Chair of Bacteriology at the McKillip Veterinary College.

THREE American Veterinary College graduates are enrolled in the corps of instructors of the Kansas City Veterinary College, namely, Drs. R. H. Harrison, F. W. Hopkins, and T. J. Turner.

HARVARD COLLEGE VETERINARY DEPARTMENT. On June 24th, at Sanders' Theatre, Boston, Mass., the commencement exercises were held. The trustees, faculties, and graduates were escorted from the college to the theatre by the National Lancers, headed by the Lieutenant-Governor.

President Eliot made a new departure in announcing and conferring the degrees in English instead of Latin as heretofore, which added much pleasure to the large audience in attendance. The following students received the degree of M.D.V.: James Munroe Armstrong, Henry Ernest Brown, Ernest Page Fuller, Fred Bryant Gage, Daniel Schafer Hays, Henry Nounse Hill, George Steven Lindenkohl, Calvert Howard Playdon, John Aloysius O'Connell, Daniel Patrick Keogh, George Edgar Fetter, George Francis Quinlan, and James Marshall.

At the commencement dinner over two thousand graduates participated and many felicitous after-dinner speeches were listened to. President Eliot announced a very important step to be adopted in the medical department after 1900, when no person will be admitted to the medical school unless he already possessed a degree in arts, letters, or science.

